# Efficacy of leflunomide combined with prednisone for the treatment of PLA2R-associated primary membranous nephropathy

**DOI:** 10.1080/0886022X.2020.1713806

**Published:** 2020-01-20

**Authors:** Yaling Guo, Xueping Wu, Lei Liu, Haifeng Zhang, Lijuan Yang, Weidong Chen

**Affiliations:** Department of Nephrology, The First Affiliated Hospital of Bengbu Medical College, Bengbu, Anhui, People’s Republic of China

**Keywords:** PLA2R, primary membranous nephropathy, prednisone, leflunomide, cyclophosphamide

## Abstract

**Objective:**

To evaluate the clinical efficacy and safety of leflunomide (LEF) combined with prednisone for the treatment of PLA2R-associated primary membranous nephropathy (PMN) and changes in anti-PLA2R antibody titers after treatment.

**Methods:**

Sixty patients with nephrotic syndrome, biopsy-proven MN and anti-PLA2R antibody positivity were included in this study conducted from December 2017 to February 2019. The patients were randomly divided into an experimental group (*n* = 30) and a control group (*n* = 30). The patients in the experimental group were treated with LEF combined with prednisone, whereas the patients in the control group were treated with cyclophosphamide (CTX) combined with prednisone. We assessed 24-h urinary protein and serum albumin levels, kidney function markers, blood lipid levels and anti-PLA2R antibody titers before and after treatment. Adverse reactions during treatment were recorded.

**Results:**

After 16 weeks of treatment, there were 2 cases of complete remission and 6 cases of partial remission in the experimental group, with a total effective rate of 26.67%. In the control group, there were 4 cases of complete remission and 8 cases of partial remission, with a total effective rate of 40% (*p* > .05). After 24 weeks of treatment, the total effective rates of the experimental and control groups were 66.67% and 76.67%, respectively (*p >* .05). There were no significant differences in 24-h urinary protein, serum albumin, kidney function marker or blood lipid levels between the two groups after treatment (*p* > .05). However, there were fewer adverse reactions in the experimental group than in the control group (*p* < .05). After treatment, serum anti-PLA2R antibody titers were clearly decreased in patients with complete remission and partial remission (*p* < .05), but these levels remained relatively high in patients without remission (*p >* .05).

**Conclusion:**

LEF combined with prednisone has a certain efficacy for the treatment of PLA2R-associated PMN and provokes few adverse reactions. A large-sample randomized double-blind controlled study with a long follow-up period is needed to verify the efficacy of LEF combined with prednisone.

## Introduction

Primary membranous nephropathy (PMN), which accounts for about one-third of cases of adult nephrotic syndrome and is also refractory nephropathy, is a common clinical pathological type of nephrotic syndrome in adults [1–3]. One-third of PMN patients have the possibility of self-healing, but 30–40% may progress toward end-stage renal disease (ESRD) within 5–15 years [4]. In China, the prevalence of PMN has doubled over the past 10 years [5], which may be related to aggravation by environmental pollution in recent years [6].

At present, the pathogenesis of PMN is still unclear, but most researchers believe it is an autoimmune disease [7,8]. A number of target autoantigens have been described, with antibodies most frequently directed against the phospholipase A2 receptor (PLA2R), which is strongly expressed in glomerular podocytes [9–11]. Anti-PLA2R autoantibodies are present in more than 75% of individuals with PMN but never in those with secondary causes of MN, other glomerular or autoimmune diseases or normal controls [7]. PMN patients with elevated serum anti-PLA2R antibody levels and PMN patients with enhanced glomerular PLA2R deposits are defined as having PLA2R-associated PMN [7]. Non-PLA2R-associated PMN might be related to other PMN target autoantigens, such as thrombospondin type-1 domain-containing 7A (THSD7A) [12–14].

Currently, general treatment for PLA2R-associated PMN patients includes the use of diuretics, angiotensin-converting enzyme inhibitors (ACEIs) or angiotensin-receptor blockers (ARBs), statins, anticoagulants, and vitamin D to alleviate the symptoms and reduce the complications associated with nephrotic syndrome. However, the main treatment is still immunosuppressants and cytotoxic drugs; rituximab acts against CD20-positive B cells, though there are no cell-specific drugs targeting PLA2R or THSD7A [15]. Different immunosuppressants have different mechanisms of action, and the optimal choice remains controversial. Leflunomide (LEF) is an immunomodulatory drug that inhibits the mitochondrial enzyme dihydroorotate dehydrogenase (an enzyme involved in *de novo* pyrimidine synthesis) [16]. LEF has been widely used to treat patients with autoimmune diseases such as systemic lupus erythematosus (SLE), rheumatoid arthritis (RA), and so on. Although there are few reports of LEF being used for the treatment of PMN, its efficacy has been shown to be marked in China [17,18]. LEF can reduce urinary protein levels and hematuria and protect renal function by decreasing the deposition of immune complexes in renal tissue. Cyclophosphamide (CTX) is a common immunosuppressive agent that suppresses the synthesis of DNA, reduces the number of lymphocytes and plays an immunosuppressive role. Clinicians have observed that combining glucocorticoids with an alkylating agent is an effective strategy for the treatment of PMN and is regarded as the gold standard of treatment [19,20].

In this study, We observed the efficacy and safety of the LEF. Prednisone combined with CTX was administered as the control, and changes in serum anti-PLA2R antibody titers were analyzed to provide a basis for the treatment and prognosis of patients with PMN.

## Methods

### Patients

This was a single-center randomized controlled study conducted from December 2017 to February 2019 that included sixty patients. All patients were diagnosed with PLA2R-associated PMN at the First Affiliated Hospital of Bengbu Medical College. This study was registered with the China clinical trial registry (ChiCTR1900027627) and approved by the Institutional Review Board of the First Affiliated Hospital of Bengbu Medical College (IRB: BYYFY-2017KY09). All participants signed the informed consent. Our inclusion criteria were as follows: (1) The age from 18 to 70 years; (2) nephrotic syndrome (24-h urinary protein ≥3.5 g and serum albumin ≤30 g/L); (3) confirmed PMN onset by renal biopsy in our hospital; (4) serum anti-PLA2R antibody positivity and/or renal tissue PLA2R antigen positivity; and (5) no use of corticosteroids or immunosuppressive agents within the last 3 months. The exclusion criteria were as follows: (1) various secondary membranous nephropathies; (2) diabetes (fasting blood glucose >6.1 mmol/L); (3) malignant tumors; (4) severe infection; (5) abnormal liver function or allergies to the treatment; (6) pregnancy or lactation; and (7) serious complications.

### Treatment

The patients were divided into an experimental group (prednisone combined with LEF) and a control group (prednisone combined with CTX) using a random numerical table method. The experimental group took 1 mg kg^−1^ d^−1^ prednisone in the morning. The dosage of prednisone was slowly reduced 8 weeks later (10% of the total dose was reduced once every two weeks when urinary protein excretion decreased) to 20 mg d^−1^; the dosage was then reduced by 5 mg every 4 weeks until it reached 10 mg d^−1^. The LEF dosage was 20 mg d^−1^, which was taken in the morning. The control group was given 0.30–0.40 g (m^2^)^−1^ CTX once every 2 weeks. The dosage of prednisone given was the same as that given to the experimental group. The serum levels of albumin, cholesterol, and kidney function markers, 24-h urinary protein excretion levels, and serum anti-PLA2R antibody titers were assessed before and after treatment.

## Observation index

Regular monitoring during treatment was performed for serum albumin, creatinine, urea, alanine transaminase, blood sugar, hemoglobin, white blood cell count, platelet count, 24-h urinary protein, systolic pressure, diastolic pressure, and adverse reactions.

### Serum anti-PLA2R antibody detection

Patient serum was examined by an enzyme-linked immunosorbent assay (ELISA) using recombinant PLA2R as the antigen: the enzyme-labeled antibody used was peroxidase-labeled rabbit anti-human IgG. The specific steps were as follows: (1) the sample and standard sample to be tested were prepared, added the enzyme reagent, and stood for 1 h at 37 °C; (2) the reaction hole was cleaned with solution for 5 times, and the developer was added, and incubated at 37 °C for 15 min; and (3) changed the color from blue to yellow and add the termination solution. We determined the absorbance of each hole at 450 nm by enzyme labeling. The anti-PLA2R antibody (IgG) provided in the kit was used to generate a standard curve, and a four-parameter fitting equation was applied to calculate the concentration of the detected antibody (concentration <14 RU/ml was negative). The kit provides negative and positive controls.

### Evaluation of therapeutic effects

Complete remission expressed as no edema symptom, 24-h urinary protein <0.3 g and normal serum albumin and serum creatinine levels. Partial remission expressed as a reduction in edema symptom and 24-h urinary protein <3.5 g or a decrease by at least 50% compared with the highest peak value. Inefficacy expressed as no improvement in laboratory results or symptoms.

### Study endpoint

Due to time constraints, the observation period of this study was 24 weeks, which was the endpoint of the study.

### Statistical analysis

SPSS 23.0 software was used for the statistical analysis. Measurement data are expressed as the mean ± standard deviation or median + IQR. The independent sample *t*-test was applied for comparisons between the two groups. Single-factor ANOVA was used for comparisons between three groups and multiple comparisons. The paired *t-*test was employed for comparisons before and after treatment. The nonparametric test was applied for non-normal distribution data. The chi-square test was used to compare count data. *p* < .05 was considered statistically significant.

## Results

### Baseline data

Sixty patients with PMN who were positive for serum anti-PLA2R antibodies, indicating nephrotic syndrome, were randomly divided into two groups. There was no significant difference in age, sex, course of the disease, blood pressure, BMI, serum albumin levels, blood lipid levels, 24-h urinary protein levels, eGFR, serum anti-PLA2R antibody titers or pathological stage before treatment between the two groups (*p* > .05) (Table 1).

**Table 1.** Basic data in the two groups.

Characteristics	Experimental group (*n* = 30)	Control group (*n* = 30)	*p*
Sex (male/female)	17/13	15/15	.61
Age (years)	49.13 ± 13.71	49.20 ± 11.59	.98
Course of disease (months)	5.05 ± 1.20	4.25 ± 0.98	.61
Systolic pressure (mmHg)	136.77 ± 19.28	132.13 ± 22.79	.40
Diastolic pressure (mmHg)	83.67 ± 8.50	82.33 ± 12.94	.64
BMI (kg/m^2^)	25.73 ± 5.09	25.05 ± 5.75	.63
Serum albumin (g/L)	23.22 ± 3.76	21.93 ± 2.83	.14
Daily urinary protein (g/24h)	6.77 ± 2.00	6.46 ± 1.57	.51
Scr (µmol/L)	70.09 ± 11.94	70.50 ± 13.97	.90
BUN (mmol/L)	5.48 ± 1.35	5.91 ± 1.31	.22
eGFR (ml/min (l.73 m^2^))	90.87 ± 6.21	90.13 ± 6.20	.65
CHO (mmol/L)	8.92 ± 2.21	8.12 ± 1.68	.12
TG (mmol/L)	3.22 ± 1.78	3.03 ± 1.23	.62
MN-II:MN-III (*n*)	24:6	23:7	.75

There was no significant difference in age, sex, course of disease, blood pressure, BMI, serum albumin, 24-h urinary protein, renal function, serum lipid levels or pathological stage between the experimental group and the control group (*p* > .05).

BMI: body mass index; Scr: serum creatinine; BUN: blood urea nitrogen; eGFR: estimated glomerular filtration rate; CHO: cholesterol; TG: triglyceride; MN-II/III: pathological stages of membranous nephropathy.

### Clinical efficacy in the two groups

**Figure 1.**
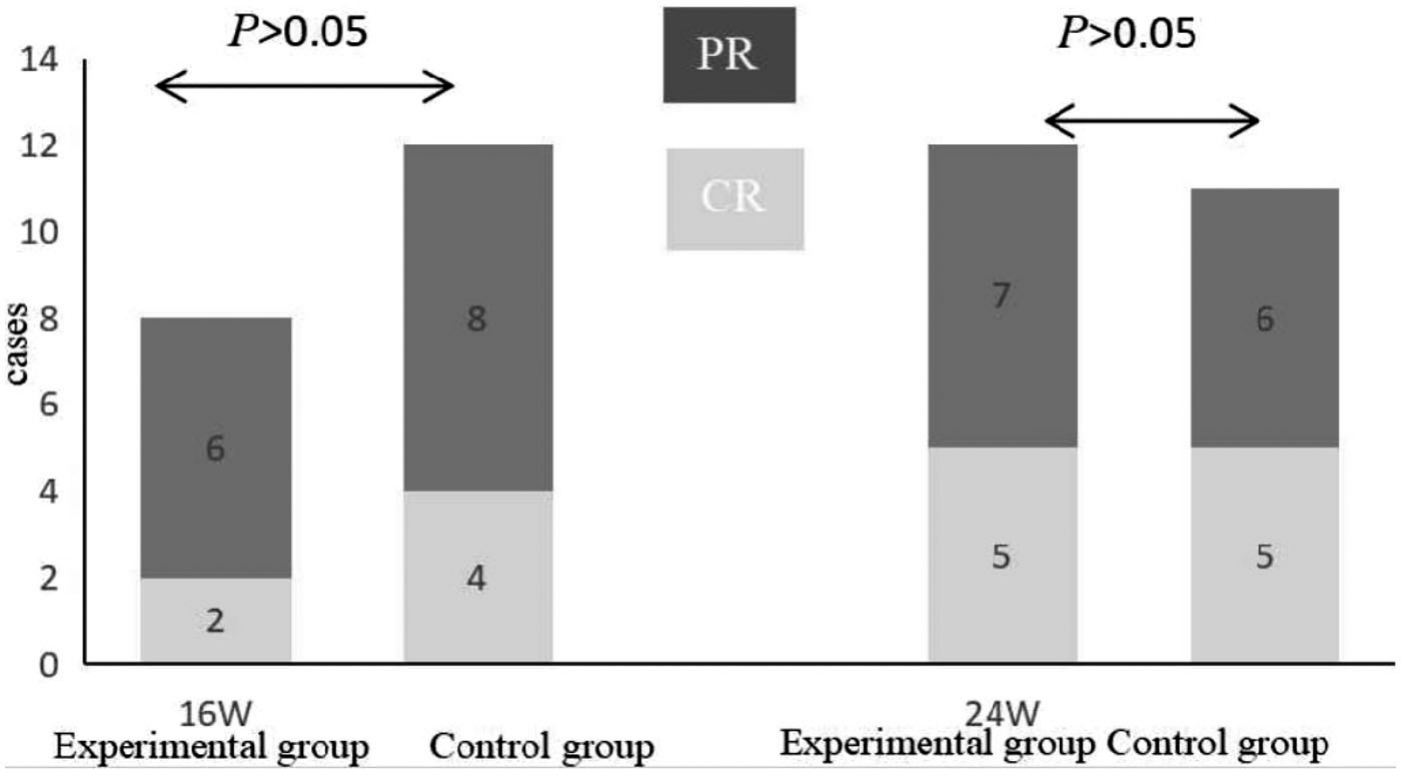
Clinical efficacy in the two groups. After treatment for 16 weeks and 24 weeks, the remission rates were similar between the two groups (*p* > .05). PR: partial remission; CR: complete remission.

After 16 weeks of treatment, the experimental group had complete remission and partial remission respectively 2 and 4 patients, with a clinical effective rate of 26.67%; the control group had complete remission and partial remission respectively 4 and 8 patients, with a clinical effective rate of 40%. After 24 weeks of treatment, the experimental group had complete remission and partial remission respectively 7 and 13 patients, with a clinical effective rate of 66.67%; the control group had complete remission and partial remission respectively 9 and 14 patients, with a clinical effective rate of 76.67%. There was no significant difference in clinical efficacy between the two groups (*p* > .05) (Figure 1).

### Comparison of 24-h urinary protein and serum albumin levels between the two groups

**Figure 2.**
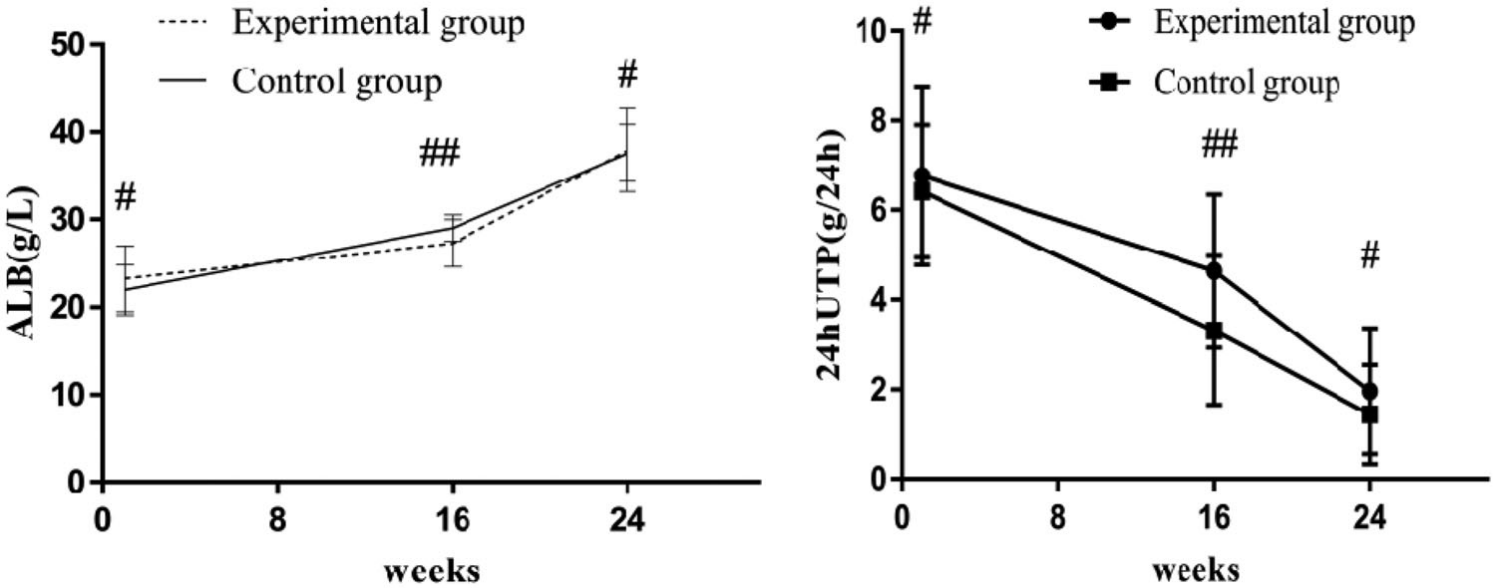
Albumin and 24-h urinary protein levels in the two groups. Before treatment, there was no significant difference in albumin or 24-h urinary protein levels between the two groups (#*p* > .05); after 16 weeks of treatment, there were significant differences in albumin or 24-h urinary protein levels between the two groups (##*p* < .05). 24-h UTP: 24-h urinary protein; ALB: albumin.

Before treatment, the 24-h urinary protein levels of the experimental and control groups were 6.99 (5.28–8.21) g/24 h and 6.38 (5.47–7.5) g/24 h, respectively; after 16 weeks of treatment, the 24-h urinary protein levels of the two groups were 4.3 (3.41–6.32) g/24 h and 3.25 (1.75–5.27) g/24 h, respectively, and the difference was statistically significant (*p* < .05); serum albumin levels were 27.29 ± 2.71 g/L and 29.03 ± 1.51 g/L, respectively, and the difference was statistically significant (*p* < .05). After 24 weeks of treatment, the 24-h urinary protein levels in the experimental and control groups were 1.95 (0.96–2.78) g/24 h and 1.37 (0.31–2.36) g/24 h, respectively, though the difference was not significant (*p >* .05), and serum albumin levels were 37.90 ± 4.76 g/L and 37.62 ± 3.25 g/L, respectively, with no significant difference (*p >* .05). After treatment, the levels of 24-h urinary protein in the two groups improved significantly and serum albumin levels were significantly increased (*p* < .05) (Figure 2).

### 24-h Urinary protein and serum albumin levels and serum anti-PLA2R antibody titers

**Figure 3.**
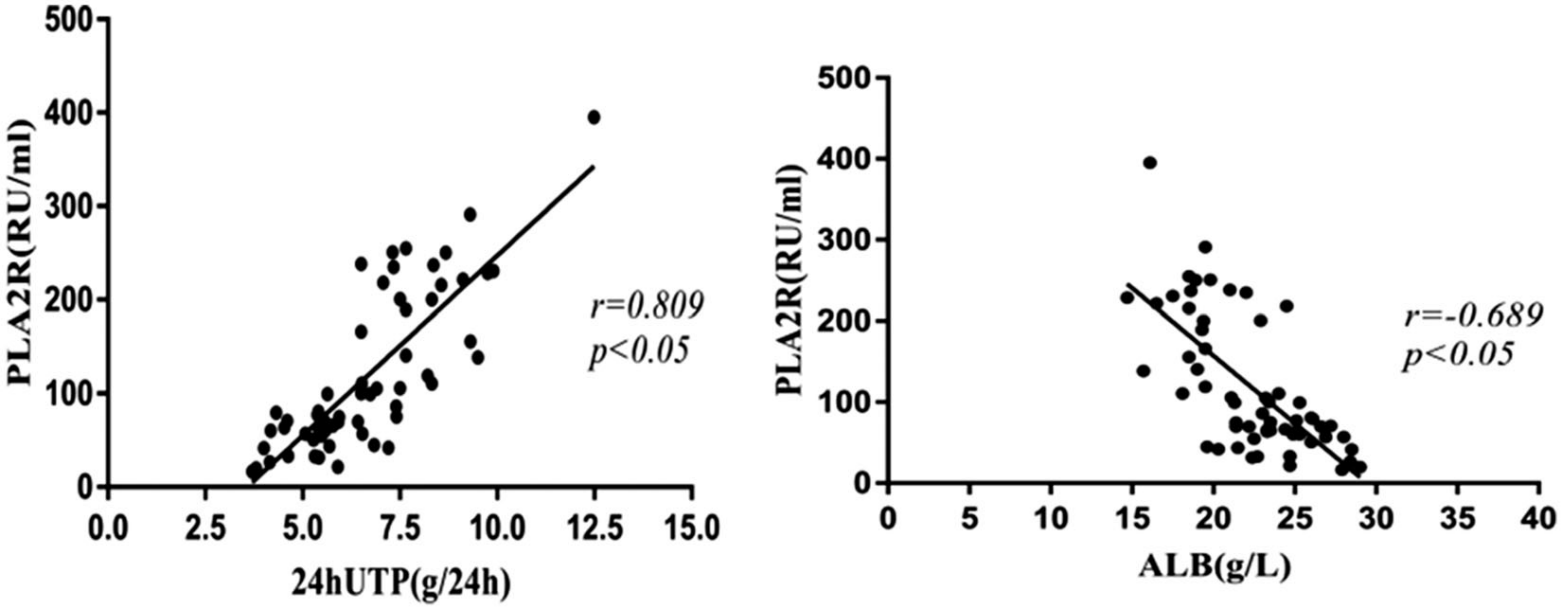
24-h urinary protein and serum albumin levels and serum anti- PLA2R antibody titers. Serum anti-PLA2R antibody titers showed a positive correlation with 24-h urinary protein levels (*p* < .05) and a negative correlation with serum albumin levels (*p* < .05). 24hUTP: 24h urinary protein; ALB: serum albumin.

Serum anti-PLA2R antibody titers were positively correlated with 24-h urinary protein levels (*r* = 0.0809, *p* < .05) and a negative correlation with serum albumin levels (*r*=−0.689, *p* < .05) (Figure 3).

### Comparison of serum anti-PLA2R antibody titers between the two groups

Before treatment, serum anti-PLA2R antibody titers in the experimental group were slightly higher than those in the control group (*p* < .05). After treatment, the titers of anti-PLA2R antibody in both groups decreased significantly comparing with those before treatment (*p* < .05) (Table 2).

**Table 2.** Comparison of serum anti-PLA2R antibody titers between the two groups.

Groups	Before treatment Serum anti-PLA2R antibody (RU/ml)	After treatment Serum anti-PLA2R antibody (RU/ml)	*P*
Experimental group	88.89 ± 7.04	50.02 ± 4.00	0.00
Control group	81.18 ± 7.70	39.78 ± 4.14	0.00
*p*	0.00	0.00	

After treatment, the anti-PLA2R antibody titers in both groups were significantly lower than those before treatment (*p* < .05).

### Serum anti-PLA2R antibody titers in patients with different prognoses

**Figure 4.**
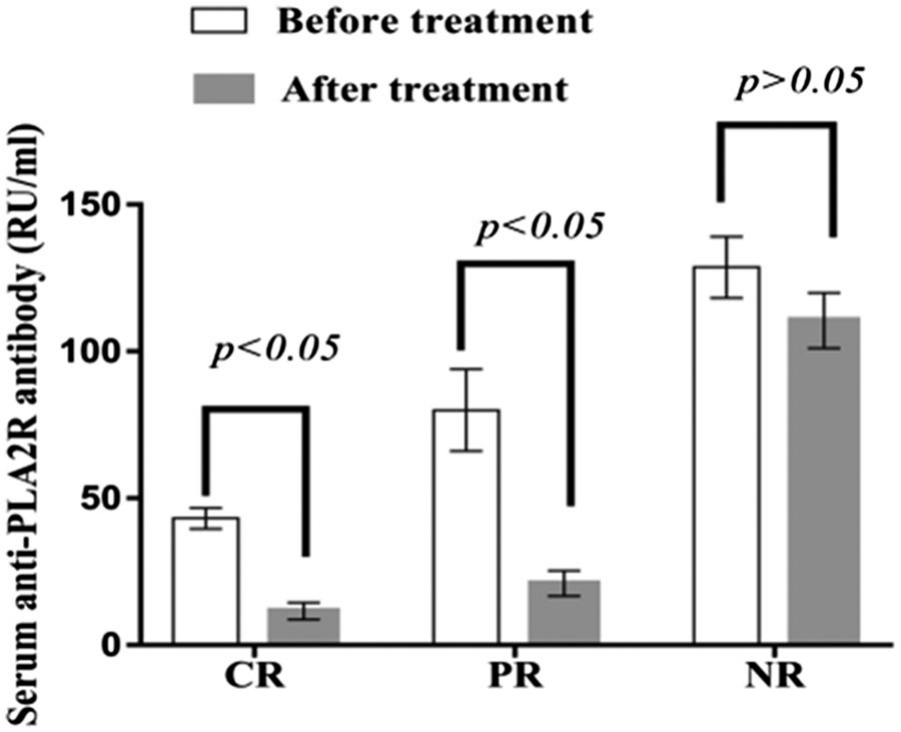
Serum anti-PLA2R antibody titers in patients with different prognoses. The serum PLA2R antibody titers in patients with complete remission and partial remission decreased significantly after treatment (*p* < .05), and there was no significant difference in the serum PLA2R antibody titers of patients with nonremission after treatment (*p* > .05). PR: partial remission; CR: complete remission; NR: nonremission.

Before treatment, serum anti-PLA2R antibody titers in patients with complete remission, partial remission and nonremission were 43.15 ± 3.56 RU/ml, 79.99 ± 13.98 RU/ml and 132.47 ± 6.47 RU/ml, respectively; after treatment, the values were 11.54 ± 2.89 RU/ml, 20.99 ± 4.32 RU/ml and 113.69 ± 5.38 RU/ml, respectively. Serum anti-PLA2R antibody titers decreased significantly in patients with complete and partial remission after treatment (*p* < .05), but there was no significant difference in the titers of patients without remission after treatment (*p* > .05) (Figure 4).

### Comparison of renal function and blood lipid levels between the two groups

**Figure 5.**
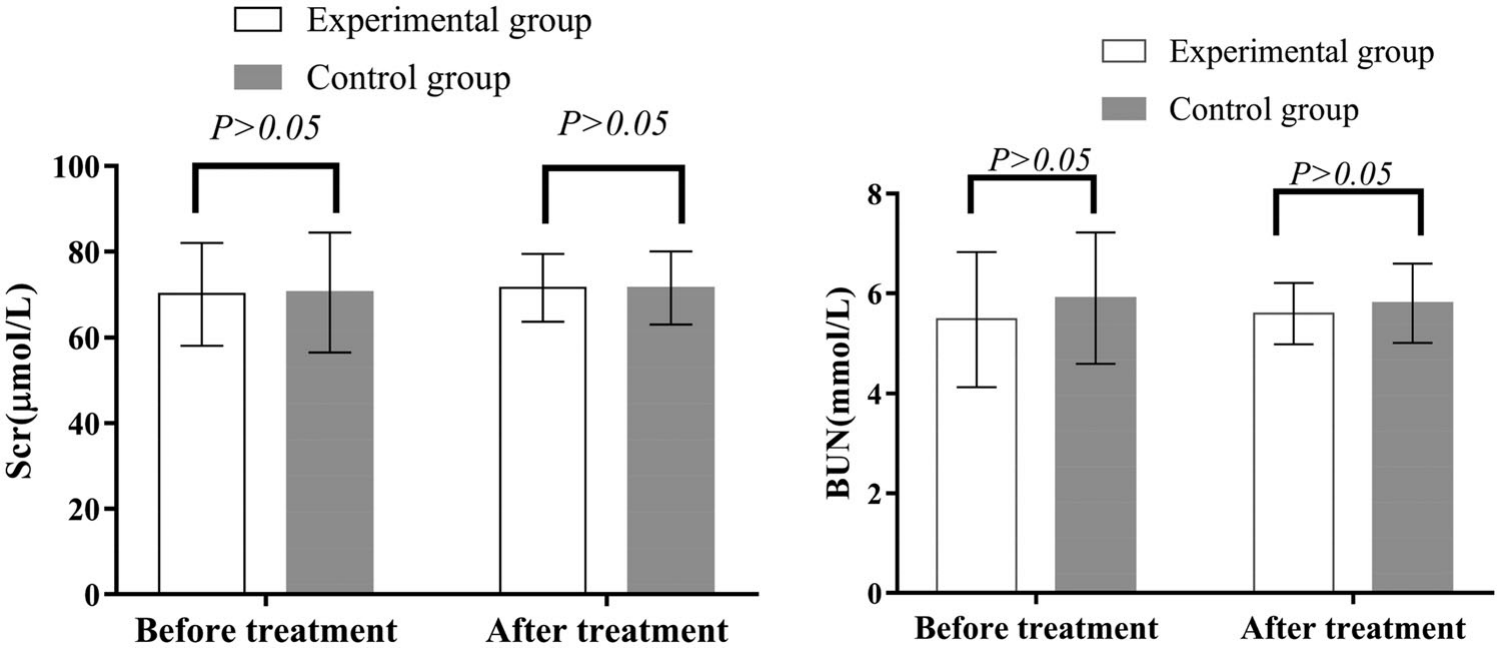
Renal function before and after treatment in the two groups. Before and after treatment, there was no significant difference in Scr and BUN between the two groups (*p* > 0.05). Scr: serum creatinine; BUN: blood urea nitrogen.

Before treatment, there was no significant difference in serum creatinine, blood urea nitrogen levels or eGFR between the two groups (*p* > .05), and no significant changes after treatment (*p* > .05) (Table 3, Figure 5).

**Table 3.** Comparison of eGFR between the two groups.

Groups	Before treatment eGFR (ml/(min) 1.73 m^2^)	After treatment eGFR (ml/(min) 1.73 m^2^)	*p*
Experimental group	90.87 ± 6.21	89.07 ± 5.19	.16
Control group	90.13 ± 6.20	89.33 ± 5.07	.15
*p*	.65	.84	

There was no significant difference in eGFR between the two groups before and after treatment (*p* > .05).

eGFR: estimated glomerular filtration rate.

Before treatment, there was no statistically significant difference in triglyceride or cholesterol levels between the two groups (*p* > .05). After treatment, the levels of triglyceride and cholesterol decreased significantly comparing with before treatment, and the differences were statistically significant (*p* < .05) (Table 4).

**Table 4.** Changes in blood lipids before and after treatment in the two groups.

		TG (mmol/L)			CHO (mmol/L)		
Group	*n*	Before treatment	After treatment	*p*	Before treatment	After treatment	*p*
Experimental group	30	3.22 ± 1.78	2.04 ± 0.82	.00	8.92 ± 2.21	4.64 ± 0.70	.01
Control group	30	3.03 ± 1.23	2.02 ± 0.40	.00	8.12 ± 1.68	4.37 ± 0.67	.01
*p*		.66	.92		.12	.13	

After treatment, triglyceride and cholesterol levels were significantly lower than those before treatment (*p* < .05). TG: triglyceride; CHO: cholesterol.

### Comparison of adverse reactions between the two groups

In the experimental group, there was 1 case of diarrhea and 1 case of infection; in the control group, vomiting occurred in 5 patients, alopecia in 6, abnormal liver function in 2, thrombocytopenia in 1, and infection in 1. There were significant differences in adverse events between the two groups (*p* < .05). All adverse reactions were relieved after symptomatic treatment.

## Discussion

Because the positive rate of the serum anti-PLA2R antibody in PMN patients is relatively high, it can be used instead of a renal biopsy as a noninvasive biomarker for the early diagnosis of PMN. Positivity for the anti-PLA2R antibody is an independent risk factor for remission and is also a major indicator of an immune response in PMN patients [21,22]. Compared with the traditional method of judging a curative effect based on the severity of proteinuria, determining changes in serum anti-PLA2R antibody titers is a more effective method of monitoring a patient’s condition and predicting the curative effect of treatment [23]. A decrease in anti-PLA2R antibody titers indicates disease improvement and immunological remission, whereas a persistently high serum anti-PLA2R antibody titer indicates that the patient’s condition has not improved [24,25]. It was reported in our other article that 7 of 164 PMN patients showed serum anti-PLA2R antibody negativity and renal tissue PLA2R antigen positivity [26]. In our study, there were only 3 such cases of the 60 patients; there was 1 case in the experimental group and 2 cases in the control group. All 3 cases were moderate-risk patients. Three patients achieved complete remission after 16 weeks of treatment. Furthermore, it is believed that the prognosis of such patients is good. We also found that patients with complete remission and partial remission had lower anti-PLA2R antibody titers before treatment than those who did not achieve remission. Moreover, patients with complete remission and partial remission had significantly lower anti-PLA2R antibody titers after treatment, whereas anti-PLA2R antibody titers remained high in patients who did not experience remission, which was consistent with the findings of a previous report [26–28]. The correlation analysis showed that the change in the anti-PLA2R antibody titer showed a positive correlation with the 24-h urinary protein level and a negative correlation with the serum albumin level. Therefore, monitoring changes in the serum anti-PLA2R antibody titers can assist in the diagnosis of PMN and reveal the recovery of immune function in patients during treatment.

In PLA2R-associated PMN patients with nephrotic syndrome or renal dysfunction, the early administration of glucocorticoids combined with immunosuppressants can reduce proteinuria and thrombosis and delay the progression of chronic renal disease; however, the choice of the appropriate immunosuppressive agent in the clinic is still controversial. Tacrolimus is an immunosuppressive macrolide with strong immunosuppressive effect. The main mechanism of tacrolimus is similar to that of cyclosporine A: suppressing the activation of T cells and the proliferation of T-helper cell-dependent B cells. However, tacrolimus is many times stronger than cyclosporine A. It has been reported that patients taking tacrolimus have a high remission rate after treatment but are prone to relapse, presenting with renal toxicity, neurotoxicity, hyperglycemia and a higher risk of tumors or infections [29–32].

It has been reported that rituximab might replace cyclophosphamide as a first-line immunosuppressive therapy in patients with PMN and nephrotic syndrome [33], yet rituximab has a high nonresponse rate and a low rate of partial remission in the treatment of these patients [34–36]. CTX is a periodic nonspecific drug that can affect the normal function of DNA and RNA, not only hindering the proliferation of cells but also suppressing immunity by damaging sensitive small lymphocytes. CTX is a classic treatment for membranous nephropathy, and its efficacy is recognized by doctors in most countries [20,37]. Nonetheless, the risk of infection increases with the long-term use of CTX, leading to leukopenia, alopecia, cancer and other serious adverse events [38,39]. For patients with PMN, the use of cyclophosphamide varies from center to center. Previously, our center reported a CTX dose of 8-12 mg/kg/per administration, given once every four weeks [40]. However, a large number of clinical observations suggest slow improvements in edema and proteinuria. We then adopted a dose according to a report of a CTX dose of 0.5–0.75 g/m^2^, given once every month initially [41], but this single dose was too large, and patients experienced adverse reactions such as nausea and vomiting. Finally, we used 0.30–0.40 g/m^2^ CTX once every 2 weeks such that the patients’ discomfort and symptoms were reduced and the accumulated time was shortened. Although our CTX therapy regimen was neither classic nor recommended by common guidelines, its efficacy was similar to that previously reported. A clinical study showed that the total remission rate of PMN after 12 months of CTX treatment was 82.1% [41]; it has also been reported that after 23 months of CTX treatment, the overall clinical remission rate is 75% [42]. In our study, the total effective rates of the CTX group after 16 and 24 weeks of treatment were 40% and 76.67%, respectively.

In China, medical insurance for relatively expensive drugs, such as tacrolimus, cyclosporine A and mycophenolate mofetil, which fall outside the scope of basic medical insurance in many areas, is lacking. In addition, some hospitals cannot monitor the plasma concentrations of cyclosporine and tacrolimus. LEF has an immunomodulatory function, inhibiting the activation of tyrosine kinases and interfering with T cell proliferation [43]. Although LEF has been used in relatively few patients with PMN, it has been clinically effective in the treatment of refractory nephrotic syndrome, IgA nephropathy, rheumatoid arthritis, SLE and refractory cytomegalovirus infection [18,^44–47^].

Leflunomide is effective for treating PMN and is a new immunosuppressive agent with good therapeutic potential. Domestic scholars have analyzed the efficacy of LEF and CTX in the treatment of PMN, and there were differences in the results. Nonetheless, most studies have suggested that LEF has the same efficacy as CTX [18,48,49]. Li et al. showed that after 3 months of treatment for PMN, the remission rate of LEF was lower than that of CTX; however, after 6 months of treatment, the complete remission rates of the LEF and CTX groups were 41.0% and 63.6%, respectively, and the nonremission rates were 20.5% and 12.1%, respectively. Since there was no statistical significance in the total remission rate between the two groups, the authors reported that LEF works slowly [48]. He et al. found that after 6 months of treatment for PMN, the total effective rates in the LEF and CTX groups were 73.08% and 69.23%, respectively, with no significant difference in the total effective rate between the two groups [49]. Yan et al. also showed that after 6 months of treatment with LEF and CTX for PMN, the total remission rates were 73.33% and 67.67%, respectively, but while the total remission rates for LEF or CTX combined with prednisone for the treatment of PMN were equivalent, LEF had a lower incidence of adverse reactions [50]. In our study, we found that remission occurred faster with CTX than with LEF but that the total clinical remission rate was similar between the two groups after 24 weeks of treatment, with no significant difference. LEF and CTX have the same effect in reducing the 24-h urinary protein level, increasing the serum albumin level and controlling blood lipid levels, with no significant effects on renal function. Furthermore, LEF has fewer side effects than CTX. Although diarrhea and infections were observed, no myelosuppression or renal damage occurred in any of the patients, which agreed with the results of previous reports [18,51]. LEF is cheap, with definite efficacy and comparatively high safety and few side effects. Thus, it is easy for patients to maintain therapy during follow-up, and there is no need to monitor plasma concentrations and adjust the dose. Accordingly, there are more PLA2R-associated PMN patients who choose LEF versus CTX in China.

As a single-center study, the number of cases were few, and the follow-up time was short. No follow-up renal function endpoints such as ESRD or a 50% decrease in the glomerular filtration rate were measured, and long-term randomized controlled trials are still needed for validation.

In conclusion, prednisone combined with LEF is a safe and effective treatment for patients with PLA2R-associated PMN. Anti-PLA2R antibody positivity can assist in the diagnosis of PMN and in monitoring the recovery of immune function in patients during treatment.
